# LRG1 Expression Is Elevated in the Eyes of Patients with Neovascular Age-Related Macular Degeneration

**DOI:** 10.3390/ijms22168879

**Published:** 2021-08-18

**Authors:** Lucia Mundo, Gian Marco Tosi, Stefano Lazzi, Grazia Pertile, Barbara Parolini, Giovanni Neri, Matteo Posarelli, Elena De Benedetto, Tommaso Bacci, Ennio Silvestri, Maria Chiara Siciliano, Stefano Barbera, Maurizio Orlandini, John Greenwood, Stephen E. Moss, Federico Galvagni

**Affiliations:** 1 Section of Pathology, Department of Medical Biotechnology, University of Siena, 53100 Siena, Italy; lucia.mundo.ml@gmail.com (L.M.); lazzi2@unisi.it (S.L.); siciliano10@student.unisi.it (M.C.S.); 2 Health Research Institute, University of Limerick, V94 T9PX Limerick, Ireland; 3 Ophthalmology Unit, Department of Medicine, Surgery and Neuroscience, University of Siena, 53100 Siena, Italy; gianmarco.tosi@unisi.it (G.M.T.); gio.neri2009@libero.it (G.N.); mposarelli@gmail.com (M.P.); elenadebenedetto1992@gmail.com (E.D.B.); osammot.iccab@gmail.com (T.B.); 4 IRCCS Sacro Cuore Don Calabria Hospital, 37024 Negrar, Italy; grazia.pertile@gmail.com; 5 Vitroretinal Unit, Sant’Anna Hospital, 25127 Brescia, Italy; parolinibarbara@gmail.com; 6 Department of Biotechnology, Chemistry and Pharmacy, University of Siena, Via A. Moro, 2, 53100 Siena, Italy; ennio.silvestri@unige.ch (E.S.); stefano.barbera@student.unisi.it (S.B.); maurizio.orlandini@unisi.it (M.O.); 7 UCL Institute of Ophthalmology, 11-43 Bath Street, London EC1V 9EL, UK; j.greenwood@ucl.ac.uk

**Keywords:** LRG1, age-related macular degeneration, AMD, VEGF, choroidal neovascular membranes

## Abstract

Leucine-rich a-2-glycoprotein 1 (LRG1) is a candidate therapeutic target for treating the neovascular form of age-related macular degeneration (nvAMD). In this study we examined the expression of LRG1 in eyes of nvAMD patients. Choroidal neovascular membranes (CNVMs) from patients who underwent submacular surgery for retinal pigment epithelium–choroid graft transplantation were collected from 5 nvAMD patients without any prior intravitreal anti-VEGF injection, and from six patients who received intravitreal anti-VEGF injections before surgery. As controls free of nvAMD, retina sections were obtained from the eyes resected from a patient with lacrimal sac tumor and from a patient with neuroblastoma. CNVMs were immunostained for CD34, LRG1, and α-smooth muscle actin (α-SMA). Aqueous humor samples were collected from 58 untreated-naïve nvAMD patients prior to the intravitreal injection of anti-VEGF and 51 age-matched cataract control patients, and LRG1 concentration was measured by ELISA. The level of LRG1 immunostaining is frequently high in both the endothelial cells of the blood vessels, and myofibroblasts in the surrounding tissue of CNVMs of treatment-naïve nvAMD patients. Furthermore, the average concentration of LRG1 was significantly higher in the aqueous humor of nvAMD patients than in controls. These observations provide a strong experimental basis and scientific rationale for the progression of a therapeutic anti-LRG1 monoclonal antibody into clinical trials with patients with nvAMD.

## 1. Introduction

Age-related macular degeneration (AMD) is one of the most common causes of visual impairment and sight loss in the elderly that may be irreversible. The majority of cases manifest with geographic atrophy, in which vision loss is associated with progressively larger regions of photoreceptor death, particularly in and around the macula. Although the atrophic form is the most frequent presentation of the disease, a significant number of patients present with an alternative pathology characterized by abnormal vascular growth in the choroid and/or retina, where vessel leakage causes localized oedema and leads to damage to the retinal tissue. Treatment of this neovascular form of AMD (nvAMD), which is responsible for around 90% of acute blindness due to AMD [1], is primarily delivered through the application of inhibitors of vascular endothelial growth factor (VEGF) such as ranibizumab and aflibercept [2,3]. However, anti-VEGF treatment efficacy is time-dependent, and it correlates strongly with visual acuity at the time of the first injection [4,5]. Nevertheless, in many patients, frequent injections of these VEGF blockers are effective in reducing vessel leakage and preventing further vessel growth, and this is typically accompanied by improvements in visual acuity. Despite this success, not all patients respond favourably to VEGF blockers, and even those that do initially respond may eventually become refractive, resulting in continuing disease progression [6].

The lack of total therapeutic penetrance indicates that factors other than VEGF contribute to pathology in nvAMD, and indeed there are ongoing attempts to target other angiogenic factors such as angiopoietin-2 (Ang2), through agents such as Faricimab, a bi-specific inhibitor of Ang2 and VEGF [7,8]. However, several other humoral mediators are altered in patients affected by nvAMD [9,10,11], and other candidate therapeutic targets continue to be sought. One such study led to the identification of leucine-rich a-2-glycoprotein 1 (LRG1). This secreted glycoprotein was found to be upregulated in the abnormal vessels of a number of mouse models of retinal vascular disease and was shown to be a contributing factor to their dysfunction [12]. In normal healthy individuals LRG1 is expressed almost exclusively by the liver from where it is secreted into the circulation and is present at low levels in plasma. The function of LRG1 under normal circumstances remains unclear, but in a variety of pathologies including cancer and diabetes LRG1 levels are significantly increased [13,14,15]. It is noticeable in such conditions that LRG1 gene expression becomes activated in endothelial cells, and there is growing evidence that the ectopic expression of LRG1 in pathological vascular beds contributes to the vascular abnormalities that are frequently observed in disease lesions [16,17].

Proteomic studies have identified LRG1 as a factor present in the vitreous and aqueous of patients with nvAMD and diabetic eye disease [18,19,20]. In healthy individuals LRG1 is almost undetectable in the eye, hence expression in the disease may contribute to the vascular pathology that accompanies both conditions. In this study we have examined the expression of LRG1 in the choroidal neovascular membranes (CNVMs) and the aqueous humor of nvAMD patients, in order to determine whether diseased retinal tissue is a source of LRG1, and if so, to ask whether treatment with VEGF blockers has any impact on LRG1 expression.

## 2. Results

### 2.1. LRG1 Is Expressed in Vessels and Myofibroblasts of CNVMs of nvAMD Patients

**Figure 1:**
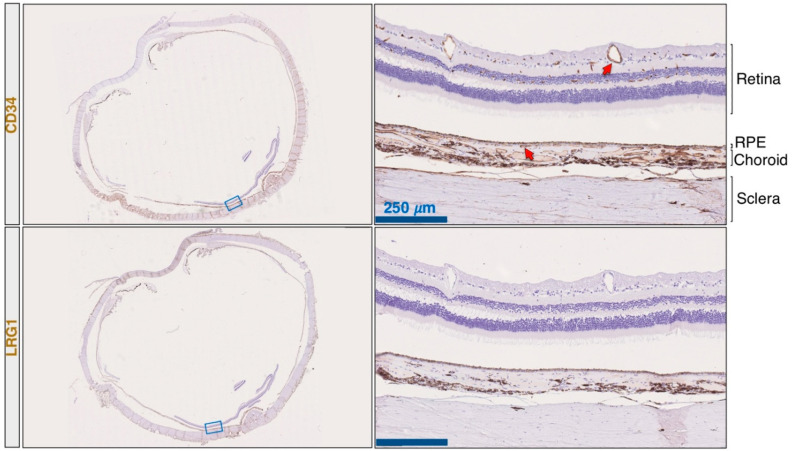
LRG1 is not expressed in normal choroidal and retinal vessels. The eye from a patient with a lacrimal sac tumor was resected, formalin-fixed, and paraffin-embedded. Serial sections were analyzed by immunohistochemical staining using anti-CD34 and anti-LRG1 antibodies. In the right panels, arrows indicate choroidal and retinal blood vessels. Left panels show entire eye sections, and blue rectangles indicate the magnification areas shown in the right panels. Scale bars: 250 μm.

**Figure A1:**
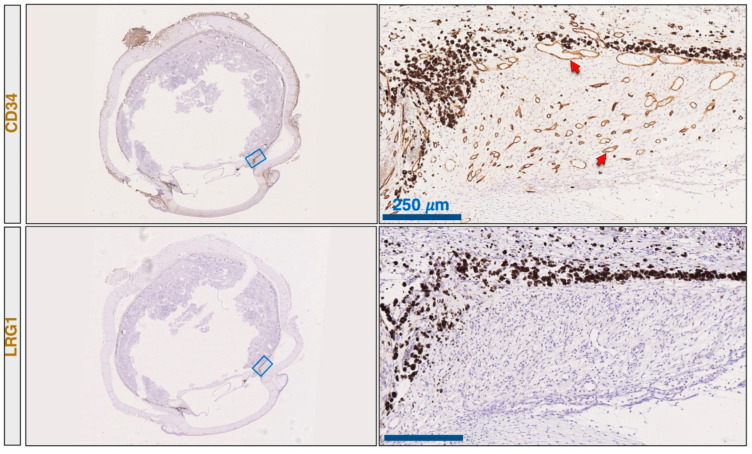
LRG1 is not expressed in normal choroidal and retinal vessels (from patient with retinoblastoma). Serial paraffin-embedded sections from human normal choroids were analyzed by immunohistochemical staining using anti-CD34 and anti-LRG1 antibodies. In the right panels, arrowheads indicate choroidal and retinal blood vessels. Left panels show entire eye sections, and blue rectangles indicate the magnification areas shown in the right panels. Scale bars: 250 μm.

To investigate the expression of LRG1 in the choroidal and retinal vessels of individuals free of nvAMD, we performed an immunohistochemical staining for LRG1 on longitudinal sections of eye specimens resected from a patient with a lacrimal sac tumor (Figure 1), and from a patient with retinoblastoma (Figure A1). CD34 staining was used to identify endothelial blood vessel cells. This analysis failed to detect any evidence of LRG1 expression in normal quiescent vessels from either retina or choroid. 

**Figure 2:**
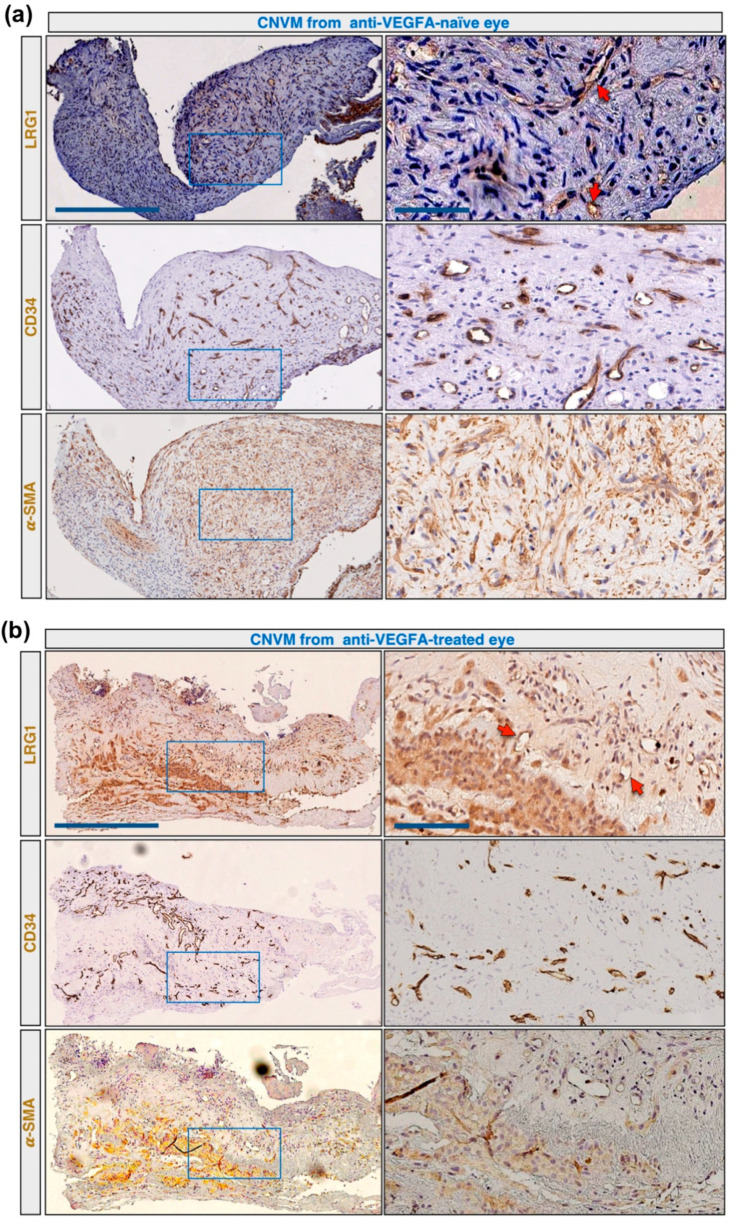
LRG1 is expressed in pathological vessels of CNVM. Serial CNV membrane sections were analyzed by immunohistochemistry using anti-LRG1, anti-CD34, and anti-α-SMA antibodies. High magnifications of stained sections displayed in the blue rectangles are shown on the right. Arrows indicate blood vessels positive for LRG1. (**a**) CNV membrane from an anti-VEGFA-naïve eye with LRG1 expression in vessels and myofibroblasts. (**b**) CNV membrane from an anti-VEGFA-treated eye with LRG1 expression in vessels. Scale bars: 250 μm; 50 μm for magnifications.

In order to assess whether LRG1 is expressed in the blood vessels of CNVMs of nvAMD patients, appropriate specimens from an anti-VEGFA-naïve and a treated eye were collected during submacular surgery for retinal pigment epithelium–choroid graft transplantation and immunostained for CD34, LRG1, and α-smooth muscle actin (α-SMA) to detect myofibroblasts. In contrast to the earlier control samples, we detected a variable expression of LRG1 in both vessels and myofibroblasts in these sections (Figure 2a,b).

**Figure 3:**
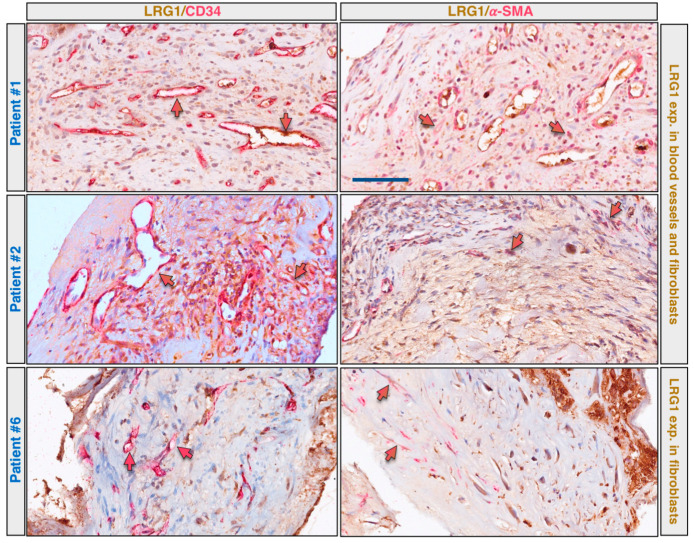
LRG1 is expressed in pathological vessels and myofibroblasts of CNVM. CNV membrane sections were analyzed by multiplex immunohistochemistry using anti-LRG1 (brown staining) and anti-CD34 (red staining), or anti-LRG1 (brown staining) and anti-α-SMA (red staining) antibody combinations. Results of three representative patients are shown. Red arrows indicate blood vessels negative for LRG1; red/brown arrows indicate blood vessels or myofibroblasts positive for both LRG1 and CD34, or LRG1 and α-SMA. Scale bar: 50 μm.

**Figure A2:**
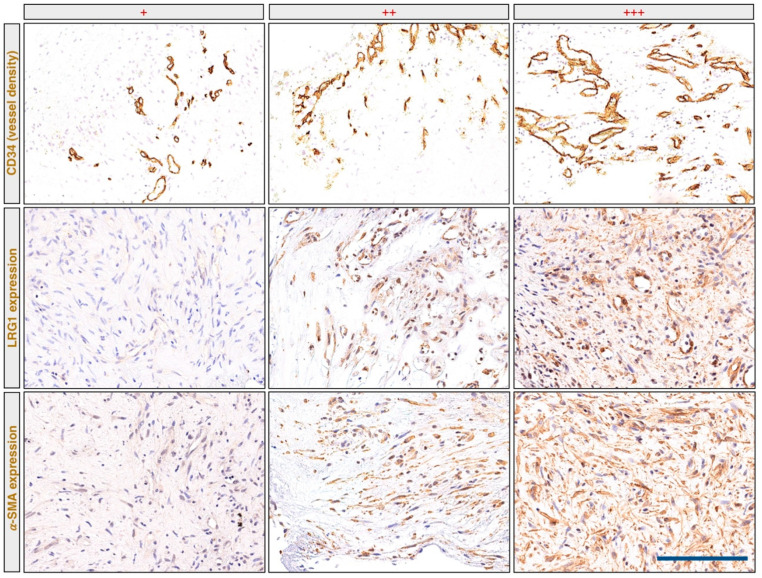
Example panels showing the scores used for vessel density, LRG1 expression, and α-SMA positivity evaluation. Paraffin-embedded sections from CNVMs were analyzed by sIHC staining using anti-CD34, anti-LRG1, or anti- α-SMA antibodies, respectively. Scale bars: 100 μm.

To extend the analysis and to evaluate better the expression of LRG1 in a cohort of different patients and cell subtypes, CNVMs from eleven patients were stained by multiplex immunohistochemistry (mIHC) for the simultaneous evaluation of the expression of LRG1 with α-SMA or CD34 (a comparative scale used for this evaluation is reported in Figure A2). Five samples were obtained from patients who underwent submacular surgery without any prior intravitreal anti-VEGF injection, while samples were also investigated from six patients who received intravitreal anti-VEGF injections before surgery. The interval between the last intravitreal injection and surgery varied from 3 months to 5 months. The patients’ characteristics and the mIHC results are reported in Table 1 and some exemplary results are shown in Figure 3.

Even though the small sample size limits the generalizability of the results, our findings suggest that LRG1 is expressed in the vessels and/or myofibroblasts of nvAMD patients at higher levels in the treatment-naïve patients.

### 2.2. Soluble LRG1 Levels Are Higher in Naïve nvAMD Patients

To further explore the expression of LRG1 in the eyes of patients with exudative AMD, we used ELISA to measure the concentration of soluble LRG1 in the aqueous humor of 58 untreated nvAMD patients (naïve nvAMD), with 51 age-matched cataract patients constituting the control group (Cntr.) (Table 2). 

**Figure 4:**
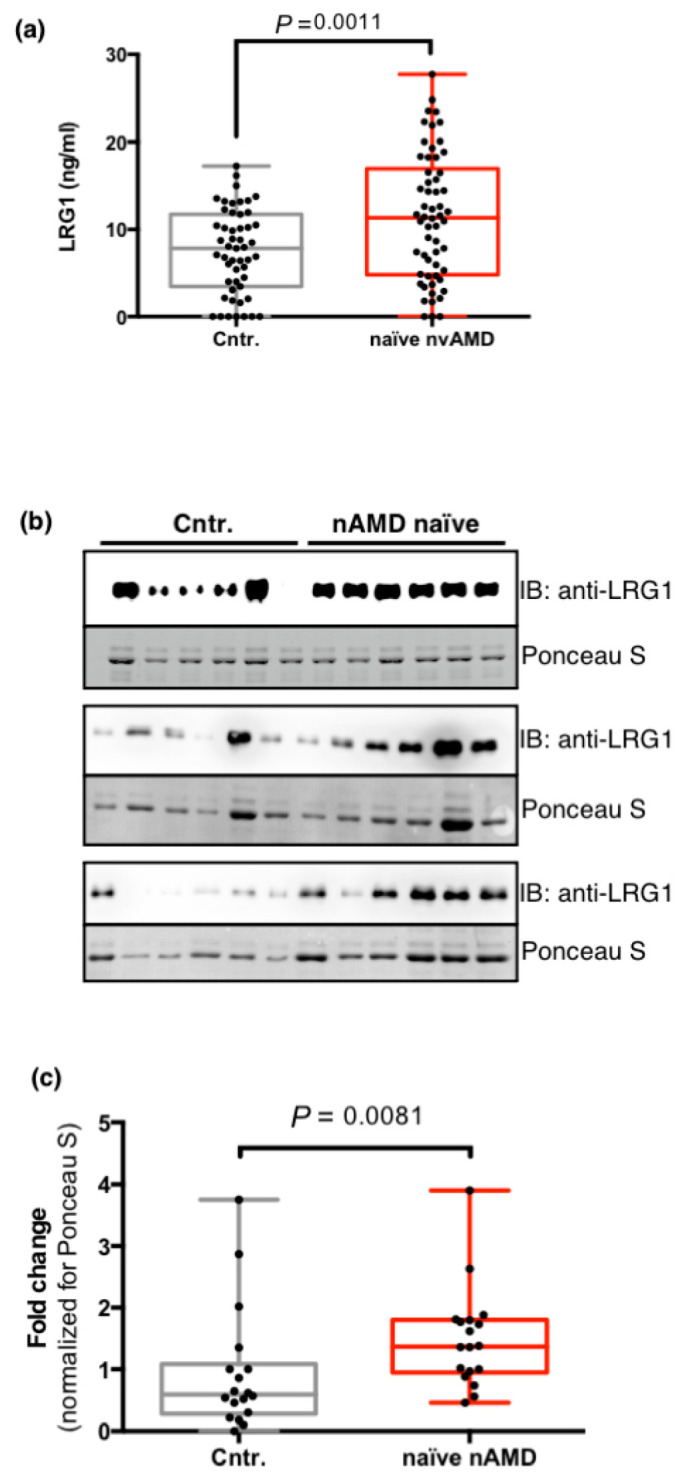
Analysis of the humor aqueous levels of LRG1. (**a**) LRG1 protein concentrations in the aqueous humor of 58 naïve nvAMD patients (nvAMD) and 51 controls (Cntr.) (described in Table 2) were determined by ELISA. Each sample was measured twice. Data are presented as box and whisker plots displaying the median, lower, and upper quartiles (boxes) and minimum–maximum (whiskers). The *P* value was calculated by a two-tailed unpaired Student’s *t*-test. (**b**) Western blot analysis of LRG1 levels in the aqueous humor of 18 randomly selected nvAMD patients and controls. Loading control is shown as Ponceau S staining. (**c**) Quantitative analysis of panel (**b**) shows a significant increase in LRG1 expression in nvAMD patients. LRG1 intensity was normalized by the intensity of Ponceau-stained proteins, and protein levels were presented as fold changes. The *P* value was calculated by a Mann–Whitney nonparametric U test.

The mean age was 76 ± 8 years in patients affected by nvAMD and 72 ± 9 in controls. The male-to-female ratio was 1:1 and 1:1.04 in patients and controls, respectively. As shown in Figure 4a, the average concentration of LRG1 was significantly higher in the aqueous humor of nvAMD patients than in controls (baseline AMD mean = 11.46 ± 7.22 ng/mL [range of variation = 0–27.74], controls mean = 7.46 ± 4.83 ng/mL [0–17.27]; *p* = 0.0011). To further support this observation, a Western blot of aqueous humor samples was performed and the intensity of LRG1 bands was quantified, confirming a higher concentration of LRG1 in the aqueous humors of nvAMD patients (Figure 4b,c). The presence of LRG1 in the control samples is consistent with a previous proteomic analysis of human aqueous humor [21].

## 3. Discussion

We report here the first study to examine LRG1 expression in nvAMD, and show using immunohistochemistry that in sections derived from treatment-naïve patients LRG1 is frequently expressed at high levels in both the endothelial cells of the blood vessels, and myofibroblasts in the surrounding tissue. Although the association of LRG1 with pathological blood vessels is well established, there is little evidence that LRG1 is expressed in fibroblasts, though there are reports that fibroblast expression is up-regulated in human disease [22,23]. LRG1 is generally considered to be constitutively secreted and, although some is likely to be sequestered locally in the extracellular matrix, a fraction may enter the vitreous, as has been noted in various proteomic studies [18,20] and some may in turn also appear in the aqueous. Consistent with a model of LRG1 expression at the site of the lesion accompanied by release into the fluidic ocular compartments, we also noted significantly higher levels of LRG1 in aqueous samples from treatment-naïve patients when compared to controls. Note that controls in this study were patients with cataracts, who were not necessarily representative of disease-free individuals, although our detection of LRG1 in these samples was consistent with published proteomic data [21]. 

In addition to the expected positive LRG1 staining of the vascular endothelium, co-staining for α-SMA confirmed that LRG1 is also present in myofibroblasts, thus defining two distinct cell types as sources of LRG1 in nvAMD. This was in stark comparison to normal retinal tissue where LRG1 staining was absent. The apparent abundance of myofibroblasts in the sections analyzed is consistent with the fibrosis that is frequently associated with nvAMD [6]. Interestingly, in other fibrotic conditions, including idiopathic pulmonary fibrosis [24] and diabetic nephropathy [15], LRG1 has been reported to drive fibrosis by modulating TGF-β signaling. Our observations raise the possibility that in nvAMD, myofibroblast-derived LRG1 may contribute in a paracrine fashion to vascular dysfunction while also acting in an autocrine manner to stimulate retinal fibrosis. Recently, in support of this hypothesis, it has been observed that LRG1 is expressed in hypertrophic scarring by myofibroblasts with high α-SMA expression, and that mechanical stretch induces LRG1 overexpression in human dermal fibroblasts [22].

Although the numbers of patients examined here are too few to allow us to draw definitive conclusions, all of those who had received anti-VEGF treatment exhibited lower, albeit variable, levels of expression of LRG1 in myofibroblasts and/or endothelial cells. It is established that most patients who received VEGF blockers exhibited varying degrees of improvement in visual acuity, attenuation of neovascular blood vessel growth and a reduction in oedema. If we assume that those investigated in our study are typical in this respect, the data suggest that at least partially correcting the retinal pathology leads to a reduction in LRG1 expression. Since LRG1 itself can drive pathological changes, a reduction in LRG1 expression would be expected in any patient who gains therapeutic benefit from anti-VEGFs. However, it may be significant that there are certain patients among those in Table 1 (#6, #10 and #11), who retain LRG1 expression at moderate or high levels in myofibroblasts. Failure to suppress LRG1 expression in these patients might be associated with ongoing disease progression as LRG1 is established as a vasculopathic factor [25]. Our data demonstrate unequivocally that LRG1 is produced locally in the nvAMD eye but this does not rule out a potential contribution from the circulating pool. Plasma LRG1 concentration in healthy controls ranges between 10 and 25 µg/mL [26,27,28] and it is possible that the aqueous humor LRG1 levels may be influenced by the diffusion of the protein from the plasma. Therefore, the aqueous humor LRG1 concentration could be influenced by VEGF levels that modulate vascular permeability and, consequently, be indirectly affected by anti-VEGF therapies. Further targeted studies, therefore, will be required to ascertain whether, in addition to locally derived LRG1, there is further contribution to the intraocular concentration from the circulating LRG1 pool.

Taking our data collectively it appears that, whilst anti-VEGF therapy is reasonably effective in reducing LRG1 expression, such treatments do not achieve the more desirable outcome of complete LRG1 suppression as we observed in normal healthy controls. Targeting LRG1 in nvAMD, either alone or in combination with anti-VEGFs, may therefore deliver a more potent therapeutic outcome than treatment with anti-VEGFs alone. In previous work we showed in mouse models of retinal vascular disease that an LRG1 blockade using a polyclonal antibody resulted in reduced leakage and vascular growth [12]. Accordingly, a fully humanized and deimmunized monoclonal antibody against LRG1 named Magacizumab, was developed that, together with the parental mouse monoclonal antibody 15C4, exhibited efficacy in a variety of in vivo and ex vivo models of retinal vascular disease and angiogenesis [22]. The data presented in the current study provide a strong experimental basis and scientific rationale for the progression of Magacizumab or its derivative Fab fragment named MagaFab, into clinical trials with patients with nvAMD.

## 4. Materials and Methods

### 4.1. Immunohistochemistry (IHC)

Single IHC staining was performed on 4 μm formalin-fixed paraffin-embedded tissue (FFPE) sections that were baked for 1 h at 60 °C. Slides were stained using the automated Ventana BenchMark ULTRA platform (Ventana, Roche diagnostic, Monza, Italy) and UltraView Universal DAB detection kit (Ventana, Monza, Italy). The sections were incubated with primary antibody for 1 hour using the mouse monoclonal antibody 15C4 against LRG1 [29] (dilution 1:100), the rabbit monoclonal EP373Y antibody against CD34 (Abcam, ab81289; dilution 1:100), and the mouse monoclonal antibody 1A4 against α-SMA (Abcam, ab7817; dilution 1:100). Immunohistochemistry (IHC) assay was carried out with appropriate positive and negative controls included in each staining run. The IHC stained sections were scanned and analyzed by a Hamamatsu NanoZoomer-XR digital whole slide scanner. Control eye specimens were resected from a patient with lacrimal sac tumor (male, 83 years) and from a patient with neuroblastoma (female, 3 years).

### 4.2. Multiplex Immunohistochemistry

To simultaneously investigate the presence of LRG1 with α-SMA or CD34, double immunostaining was performed using Ventana Discovery XT ULTRA automated staining system (Roche diagnostic, Monza, Italy). All steps were performed inside the instrument, from deparaffinization to counterstaining. Multiplex immunohistochemistry (mIHC) assay was carried out with appropriate positive and negative controls included in each staining run. A standard antigen retrieval method was employed with EXPrep and CC1 (Ventana Medical Systems) buffers. The sections were incubated with primary antibody according to the manufacturer’s instruction. The color assignment was brown for LRG1, and red for CD-34 and α-SMA. The chromogenic detection was applied by using Ultra View Universal DAB Chromogen kit (Ventana, Monza, Italy) and Ultra View Universal Alkaline Phosphatase Red kit (Ventana, Monza, Italy). The mIHC-stained sections were scanned and analyzed by a Hamamatsu NanoZoomer-XR digital whole slide scanner.

### 4.3. Subjects

CNVMs were collected from patients during submacular surgery for retinal pigment epithelium-choroid graft transplantation, formalin-fixed, embedded in paraffin, and sectioned. Briefly, following complete vitrectomy and internal limiting membrane peeling, iatrogenic retinal detachment and a 180° retinotomy were performed to gain access to the subretinal space. CNVMs were then removed and the retinal pigment epithelium–choroid patch transplantation performed. Laser retinopexy was performed to reattach the retina. The prospective observational study of LRG1 levels in the aqueous humor comprised 58 patients affected by active choroidal neovascularization (CNV) secondary to AMD. All eyes were examined at the Ophthalmology Unit of the Department of Medicine, Surgery and Neuroscience, Siena University Hospital, Siena, Italy, after approval from the institutional review board. The research adhered to the principles of the Declaration of Helsinki. Patients were treated after being informed of the nature, purpose, implications and risks of the treatment and after having signed a consent form. The nvAMD diagnosis was confirmed by fluorescein angiography, indocyanine green angiography and spectral domain optical coherence tomography. None of the patients had received any previous treatment for nvAMD, nor had they undergone any previous ophthalmic surgery, except cataract removal. Cataract surgery had to have been performed at least 9 months prior to inclusion. Controls were constituted by age-matched patients undergoing cataract surgery. The exclusion criteria for controls were any ocular disease except cataracts and any previous ophthalmic surgery. Diabetes mellitus was an exclusion criterion for both patients and controls.

### 4.4. Aqueous Humor Sample Collection

Anterior chamber taps were performed in the operating room prior to each intravitreal injection (patients) and before cataract surgery (controls) to obtain aqueous samples. A 30-gauge needle was inserted into the anterior chamber and 0.1 mL of aqueous was collected, centrifuged to remove cells and debris, aliquoted and frozen at −80 °C until analysis.

### 4.5. Assessment of the LRG1 Level in the Aqueous Humor

The LRG1 concentrations were measured by sandwich enzyme-linked immunosorbent assay (ELISA). Briefly, MaxiSorp immunoplates (Thermo Fisher Scientific, Waltham, MA, USA; #10547781) were coated overnight at 4 °C with 20 μg/mL of 15C4 mouse anti-LRG1 mAb in 0.2M NaCO3/NaHCO3 buffer pH9.4 (Sigma-Aldrich, Milano, Italy; #C3041). The 25 μL of samples and serial dilutions of human recombinant LRG1 as standard were diluted to 50 μL with a wash buffer (0.1% *v*/*v* Tween-20/PBS) and left to absorb in the wells overnight at 4 °C. Detection was performed with anti-LRG1polyclonal antibody (1:500; Sigma-Aldrich; #HPA001888), Biotinylated anti-rabbit IgG (1:40,000; Sigma-Aldrich; #B8895), Horseradish Peroxidase (HRP)-conjugated Streptavidin (1:200; Thermo Fisher Scientific; #S911), and Substrate Reagent kit (R&D Systems, Minneapolis, MN, USA); #DY999). The absorbance was measured at 450 nm with a 540 nm wavelength correction on a VersaMax microplate reader and a four-parameter logistic curve-fit was used to fit the standard curve. 

### 4.6. SDS–PAGE and Western Blotting

Proteins in the aqueous humor were separated by SDS–PAGE. Gels were transferred onto a Hybond-P PVDF membrane (GE Healthcare, Milwaukee, WI, USA) and stained using Ponceau S solution (Sigma-Aldrich; #P7170). Blots were probed with 15C4 mAb followed by horseradish peroxidase (HRP)-conjugated secondary antibodies (GE Healthcare), chemiluminescence was detected by ImageQuant LAS 4000 (GE Healthcare) and densitometry was performed using ImageQuant TL image analysis software (GE Healthcare).

### 4.7. Statistical Analysis

The data analysis was performed using Prism 6 statistical software (GraphPad Software Inc., San Diego, CA, USA). The data were presented as box and whisker plots displaying median, lower and upper quartiles and the minimum–maximum. The data distribution was tested using D’Agostino & Pearson and Shapiro Wilk tests, and differences in the protein concentrations among the groups were estimated by two-tailed unpaired Student’s *t*-test or the nonparametric Mann–Whitney U test, as appropriate. Probabilities of less than 0.05 were considered significant.

## Figures and Tables

**Table 1:** Demographics, clinical and immunohistochemical characteristics of patients subjected to submacular surgery, the CNVM of which were analyzed by immunohistochemistry for LRG1 expression. “Anti-VEGF” indicates if the patient received intravitreal anti-VEGF injection before the surgery.

Patient	Age Range	Sex	Anti-VEGF	Diabetes	α-SMA Positivity *	Vessel Density *	LRG1 Expression in Myofibroblasts *	LRG1 Expression in Vessels *
#1	80–90	M	No	Yes	++	+++	+++	+++
#2	70–80	M	No	No	++	+++	+++	+++
#3	80–90	F	No	No	++	+	+++	−
#4	60–70	M	No	No	++	+++	+++	+++
#5	70–80	F	No	No	+	+++	+	+++
#6	60–70	F	Yes	No	++	++	+++	+
#7	80–90	F	Yes	Yes	+++	+	+	+/−
#8	60–70	M	Yes	No	+	+	+	−
#9	70–80	F	Yes	No	+	+	−	+
#10	60–70	F	Yes	No	++	+	++	−
#11	70–80	M	Yes	No	++	++	++	+/−

* α-SMA positivity, vessel density and LRG1 expression were evaluated through α-SMA, CD34 and LRG1 immunostaining, respectively, and plus (+) and minus (−) signs refer to the quantification level. A comparative scale used for the evaluation is reported in Figure A2. Age range is reported to anonymise the data.

**Table 2:** Comparison of patients with exudative AMD and control group and clinical characterization of patients.

	Exudative AMD Group, *n* = 58	Control Group, *n* = 51
Age, years, mean ± SD	76 ± 8	72 ± 9
Sex, M:F	1:1	1:1.04
Eyes	58 eyes;	51 eyes;
	46.5% right	55% right
Hypertension, *n*		
Yes	39	34
No	19	17
Dyslipidemia, *n*		
Yes	32	19
No	26	32

## Data Availability

The data presented in this study are available on request from the corresponding authors.
